# Bile Acids in Autoimmune Liver Disease: Unveiling the Nexus of Inflammation, Inflammatory Cells, and Treatment Strategies

**DOI:** 10.3390/cells12232725

**Published:** 2023-11-29

**Authors:** Tianhao Zhou, AbdiGhani Ismail, Heather Francis

**Affiliations:** 1 Division of Gastroenterology and Hepatology, Department of Medicine, Indiana University School of Medicine, Indianapolis, IN 46202, USA; zhouv@iu.edu; 2 Department of Medicine, Indiana University School of Medicine, Indianapolis, IN 46202, USA; abisma@iu.edu; 3 Department of Research, Richard L. Roudebush VA Medical Center, Indianapolis, IN 46202, USA

**Keywords:** ASBT, bile acids, FXR, GPBAR1, liver diseases

## Abstract

As bile acids not solely play an essential role in nutrition absorption, but also in regulating metabolic functions as well as immune response, bile acids and their signaling pathways are increasingly acknowledged as potential therapeutic targets in the context of chronic liver diseases. Bile acid receptors such as G protein bile acid-activated receptor 1 and farnesoid X receptor are expressed in different immune cells engaged in innate immunity. Recently, a series of studies have revealed distinct functions of bile acids and bile acid receptors within the adaptive immune system. In addition, a variety of molecules targeting bile acid receptors and transporters are currently in advanced stages of clinical development. Autoimmune liver diseases including conditions like primary biliary cholangitis, primary sclerosing cholangitis, and autoimmune hepatitis can lead to chronic inflammation, fibrosis, and even cirrhosis and liver failure. In this review, we focus on the role of bile acids in the inflammatory aspects of autoimmune liver diseases.

## 1. Bile Acid Synthesis and Circulation

### 1.1. Bile Acid Synthesis

Bile acids (BAs) are end-products of cholesterol metabolism. They are exclusively synthesized in the liver by two different pathways which involve at least seventeen different enzymes. The classical or neutral BA pathway is responsible for about 90% of the total BA production and is initiated by 7α-hydroxylation of cholesterol catalyzed by cholesterol 7α-hydroxylase (CYP7A1), which is the rate-limiting enzyme for all BA production [1,2]. On the other side, the alternative or acidic pathway comprises the remaining 10% of BA synthesis, which is initiated by the mitochondrial enzyme (sterol-27-hydroxylase, CYP27A1) via oxidation of cholesterol to 25-Hydroxycholesterol 7α-hydroxylase (CYP7B1) and involves the synthesis of acidic intermediate metabolites [1,2].

Cholic acid (CA) and chenodeoxycholic acid (CDCA) are the direct result of the BA synthesis pathways and are referred to as primary BAs (PBAs). Newly synthesized PBAs are further conjugated by hepatocytes with hydrophilic molecules such as glycine or taurine to increase solubility in bile [3]. In humans, most BAs are glycine-conjugated, whereas the remaining BAs are tauro-conjugated; however, BAs of mice are almost all tauro-conjugated, with a small portion using glycine conjugation [4]. In both humans and mice, CA can be oxidized and subsequently 7α-dehydroxylated to deoxycholic acid (DCA). CDCA can be converted to hyocholic acid or ursodeoxycholic acid (UDCA). UDCA can be 7α-dehydroxylated to lithocholic acid (LCA), which can be further hydroxylated to hyodeoxycholic acid (HDCA) [5]. However, about 35% of the total BA pool in mice is converted from CDCA to tri-hydroxylated muricholic acids (MCAs) by the Cyp2c70 enzyme, which is exclusively present in mice, but not human livers [6]. Several recent and extensive reviews have effectively presented both BA synthesis and BA pathways in rodents and humans [7,8,9].

### 1.2. Enterohepatic Circulation

Conjugated BAs are actively excreted by the hepatocytes into bile canaliculi, transported via the bile ducts, and stored in the gallbladder. When a meal is eaten, cholecystokinin triggers gallbladder contraction and the disposal of bile into the intestinal lumen, where it functions as an emulsifier for dietary fats and other lipophilic substances [4,10]. The transportation process of BA is highly regulated and essential to maintain BA homeostasis. About 95% of BAs are transported to the liver back from the intestine through the enterohepatic circulation via various BA transporters primarily located on ileal enterocytes (organic solute transporter α/β (OSTα/β), apical sodium-dependent bile salt transporter (ASBT)), hepatocytes (organic anion transporting polypeptide (OATP), and Na^+^-taurocholate co-transporting polypeptide (NTCP)) [11,12]. Briefly, BAs are reabsorbed at the terminal ileum via ASBT, which can cotransport Na^+^ with BA at the apical membrane of enterocytes. After binding to BA-binding protein (IBABP), OSTα/β and multidrug resistance-associated protein 3 (MRP3) located on the basolateral side of enterocytes mediate efflux of BAs and play a key role in the portal circulation of BAs [13]. Subsequently, the BAs are transported back to the liver via the portal vein mainly by NTCP and to a lesser degree by OATP1, which are both localized to the basolateral membrane of hepatocytes. The remaining 5% of BAs are excreted through feces and replaced by newly synthesized BAs via BESP in the liver. The production of enterohepatic circulation (~500 mg/day) accounts for about 90% of actively metabolized cholesterol in the body, and the biosynthesis of steroid hormones comprises the remainder [10]. Typically, conjugated BAs may travel this route 6 to 8 times a day in a human [9]. BA concentration is tightly regulated to prevent the accumulation of highly toxic BAs, maintain metabolic homeostasis in the liver, and exhibit anti-inflammatory properties within normal physiological circumstances [5]. Recent studies have also provided broad insights into BA circulation and BA-dependent microbiome via integrative multi-omics analysis in human liver patients and mouse models [14,15,16]. In addition, cholehepatic shunting also occurs between hepatocytes and cholangiocytes within the liver and will be discussed later in Section 3.

## 2. Autoimmune Liver Diseases (AILDs)

Primary biliary cholangitis (PBC), primary sclerosing cholangitis (PSC), and autoimmune hepatitis (AIH) are all chronic diseases known to cause varying levels of liver dysfunction through complex genetic, environmental, and autoimmune interactions. While these pathologies have marked similarities, including possible progression to liver failure and cirrhosis, there are also some key differences.

### 2.1. Primary Biliary Cholangitis—PBC

PBC primarily affects intrahepatic bile ducts, leading to cholangiocyte injury, cholestasis, and occasionally end-stage cirrhosis [17]. The pathophysiology is still incompletely understood, but damage likely occurs through a combination of genetic factors (with HLA DRB1/HLA DQB1 and several other MHC class two molecules being possible inciting factors) [18], environmental factors (recurrent urinary tract infection (UTI) with *Escherichia coli* inducing autoimmunity or even xenobiotics causing molecular mimicry and resultant immune-mediated damage) [19,20], and autoimmune interactions with antimitochondrial antibody production and T-lymphocyte interactions with PDC-E2 (E2 component of mitochondrial pyruvate dehydrogenase complex) [21]. PBC is commonly seen in middle-aged women that are in peri-postmenopausal age, prevalence per 100,000people in 2015 was 24.4. The prevalence of PBC is higher in those of European backgrounds, and in the United States mortality rates have been between 9 to 14%, with age, treatment with UDCA, race, and presence of cirrhosis being factors that affected those percentages [22,23]. Diagnosis of PBC is typically considered when patients have characteristic unexplained liver function test elevations (alkaline phosphatase), elevated antimitochondrial antibody levels in combination with certain clinical features such as jaundice, pruritus, and even complications of cirrhosis if the disease is advanced [21]. Imaging with magnetic resonance cholangiopancreatography and/or liver biopsy can also often help with diagnosis but is not needed in confirming whether a patient has PBC.

Many recent studies have shown that canal of hearing loss in early-stage PBC can promote toxic BA accumulation and subsequent liver injury [24]. In late-stage PBC mouse models, Kennedy et al. showed that secretin treatment promoted hepatic BA efflux, modified BA composition, and restored bicarbonate and mucin secretion [25]. In addition, hydrophobic BAs can reduce AE2 expression in small cholangiocytes by induction of ROS, which can enhance chronic bile duct inflammation in PBC [26]. Currently, there are only two approved therapies for PBC, UDCA and obeticholic acid (OCA) [17]. UDCA is a hydrophilic BA that is thought to have anti-inflammatory effects through increased hydrophilicity of accumulating BAs, causing less BA-induced cell damage [17]. OCA is a BA that is thought to treat PBC through interactions/agonist activity with Farsenoid X-receptor (FXR), a protein that is involved in BA homeostasis [27].

### 2.2. Primary Sclerosing Cholangitis—PSC

Like PBC, PSC involves fibrosis/destruction of both intra- and extrahepatic bile ducts that can lead to stricturing and eventual cirrhosis. Although the pathogenesis of PSC is not completely clear, some genetic HLA associations are suspected like PBC [28]. In addition to cholangiocyte-mediated inflammatory cytokine and T lymphocyte activation [29], it is also theorized that gut microbes can migrate and translocate via portal circulation to the liver to cause an abnormal cholangiocyte response leading to cholangiocyte senescence [30]. More recently, long non-coding RNA H19 that is predominantly expressed by cholangiocytes has been thought to be involved in pathogenesis through the accumulation of conjugated BAs and promotion of cholestasis/fibrosis; however, studies are still exploring the role of this gene [31].

PSC is more commonly seen in men (aged 30–50) up to 60% compared to women, and interestingly up to 70% of these patients have concomitant irritable bowel disease/ulcerative colitis [32]. Overall, the prevalence of PSC in North America ranges from 0.5 to 1.3 per 100,000 people. Clinical features of PSC vary, like PBC, pruritus, fatigue, and jaundice are common. Abdominal pain, hepatomegaly, and acute bacterial cholangitis also can occur due to bile duct stricturing [21]. Diagnosis of PSC is made when there is typical cholestatic liver injury and clinical features, in combination with the characteristic structuring/dilatation of intra- and extrahepatic bile ducts seen on Magnetic Resonance Cholangiopancreatography (MRCP). Of note, perinuclear staining pattern (pANCA) levels can be elevated; however, this is not specific for PSC [33]. The prognosis of PSC is typically worse than PBC, with US studies showing estimated 10-year survival close to 60–65%, with cholangiocarcinoma (CCA) and portal hypertensive associations being common complications [32]. Unfortunately, no definitive medical management has been found to be truly effective in PSC. The usage of UDCA/OCA as well as clinical trials with antifibrotics have been used as adjuncts or bridges to liver transplantation and will be discussed below [32].

### 2.3. Autoimmune Hepatitis—AIH

AIH, as the name suggests, is an autoimmune hepatitis that is theorized to occur in genetically pre-disposed individuals (DRB1 allele variants and tumor necrosis factor-α inducible protein 3 (TNFAIP3) gene nucleotide mutations and others), who are thought to be triggered by environmental factors such as viruses and circulating autoantigens that lead to an immune-mediated response resulting in hepatocyte damage [34]. Multiple theories have been presented yet the exact pathogenesis is unclear. AIH is a relatively rare disorder, with epidemiology data in Europe showing the prevalence of 14.3 per 100,000 people, with women being more affected at an almost 4:1 ratio [35]. The age afflicted ranges from childhood up until adulthood (50s/60s) depending on the subtype. Clinical features of AIH are different from PBC/PSC in that it may present in a more fluctuating pattern, with long periods of asymptomatic disease [34,36]. Due to this, patients may have no symptoms at all, while late-presenting patients may have the typical jaundice, ascites, and hepatomegaly seen in liver failure. Diagnosis is based on scoring systems that consider autoantibodies present (including anti-smooth muscle ab), immunoglobulin G (IgG) levels, liver function tests, and other factors [34]. Steroids combined with azathioprine are generally the first-line treatment to induce immunosuppression and prevent progression to cirrhosis. However, despite this, the 10-year cumulative death rate is close to 33% [35,36].

Features of typical AIH do not necessarily present clinically the same as PBC and PSC. As mentioned above, fluctuating presentations including generalized fatigue, anorexia, jaundice, and features of advanced cirrhosis can also be common. Pruritus, which can be often a hallmark of cholestatic liver diseases such as PBC/PSC is not a diagnostic criterion for typical AIH [37]. However, there are “overlap syndromes” with both PBC and PSC in which the AIH clinical features are pre-dominant, but cholestatic features are still present. In the overlap syndromes, bile duct injury which can be a clinical or histological finding, and the resultant accumulation of toxic BAs and cholestasis shares some commonality with PBC/PSC [37]. BA metabolism and gut microbiome interactions have also been studied as a possible pathophysiologic pathway in all three autoimmune liver diseases [38,39,40].

## 3. Interactions between BAs and Cholangiocytes

Cholangiocytes regulate bile composition and bile flow via the absorption of BAs, amino acids, and glucose, as well as the secretion of Cl^−^, HCO_3_^−^, and water [41]. Cholehepatic shunting occurs when BAs are recycled between hepatocytes and cholangiocytes. During this process, a portion of the bile is absorbed by ASBT in cholangiocytes, discharged into the periductular capillary plexus via OSTα/β and MRP3, and returned to hepatocytes, which leads to increased bile flow and bicarbonate-rich choleresis (bicarbonate umbrella) [42]. Inhibition of biliary ASBT has been shown to alter the BA pool in a mouse model of PSC [43]. BAs could potentially induce hypercholeresis during cholehepatic shunting by directly activating transmembrane member 16A (TMEM16A)-mediated Cl^−^ channel activity in both human and mouse cholangiocytes [44].

G protein bile acid-activated receptor 1 (GPBAR1), formerly recognized as Takeda G protein receptor (TGR5), is a G-protein coupled BA receptor that is highly expressed in numerous organs including the liver and intestine. It plays an essential role in regulating metabolic functions and immune responses [45]. While FXR is predominantly expressed in hepatocytes in the liver, GPBAR1 is mainly found in cholangiocytes. The activation of GPBAR1 triggers the cAMP-regulated CFTR (cystic fibrosis transmembrane conductance regulator) chloride channel, which promotes bicarbonate and chloride secretion into bile. This secretion process forms the bicarbonate umbrella and prevents liver damage from the toxicity induced by BAs [46]. Interestingly, GPBAR1 localizes to the apical membrane within non-ciliated cholangiocytes and inhibits ERK signaling which promotes proliferation via increased cAMP levels. However, within ciliated cholangiocytes, GPBAR1 localizes to the cilia and operates to suppress proliferation via decreased cAMP levels [46]. Besides GPBAR1, sphingosine 1-phosphate receptor 2 (S1PR2) is another G-protein coupled BA receptor that has been identified in cholangiocytes. The activation of S1PR2 plays an important role in cholangiocyte proliferation when prompted by BAs during cholestatic conditions. It has been shown that inhibition of S1PR2 activation significantly inhibited taurocholic acid (TCA)-induced cholangiocyte proliferation and migration via an ERK1/2-dependent mechanism [47,48].

## 4. Crosstalk between BAs and Immune Cells

Inflammation is a hallmark of chronic liver diseases, which contributes to the progression of liver fibrosis. There is a growing consensus that BAs have both pro- and anti-inflammatory actions through different nuclear and cell surface BA receptors in the intestine, as well as the liver [49]. Among these receptors, FXR and GPBAR1 are the most extensively studied and understood. FXR is a nuclear receptor that is preferentially activated by PBAs, and GPBAR1 is a cell membrane G protein-coupled receptor that is activated by secondary bile acids.

### 4.1. BAs and Immune Cells in Innate Immune System

The expression of FXR and GPBAR1, as well as other BA receptors (such as VDR and LXRs) has been extensively studied in cells of the innate immune system, such as circulating monocytes, macrophages, dendritic cells (DCs), natural killer (NK) cells, as well as NKT cells [50]. FXR can play a trans-repression role on inflammatory cytokines in monocytes and macrophages by both SHP (small heterodimer partner)-dependent and independent mechanisms. SHP plays a vital role in the negative feedback regulation of BA synthesis, whose activation is strongly induced by FXR. SHP has been identified as a transcriptional repressor at the promoter of FXR target genes, and it can hinder the binding of AP1 and p65 to inflammatory genes in cholestatic mouse models [51,52]. With the absence of SHP, FXR is recruited to inducible nitric oxide synthase (iNOS) and interlukin-1 β (Il-1β) promoters under agonist binding to stabilize the NCoR1 complexes and trans-repress these two genes [4,53]. In the context of DCs and NKT cells, FXR activated by INT-747/OCA inhibits the production of tumor necrosis factor alpha (TNF-α) and reduces the differentiation and activation of intestinal DCs [54]. FXR activation in liver NKT cells results in a SHP-mediated inhibition of osteopontin production, which is a potent pro-inflammatory mediator [53]. In addition, mast cells (MCs) infiltrate the liver during cholestatic liver injury and trigger biliary damage. Recently, it has been shown that MCs infiltrate the intestine and induce intestinal inflammation and cholestasis through FXR/FGF15 (fibroblast growth factor (FGF15) signaling [55]. Further, inhibition of biliary ASBT decreases cholestatic phenotypes in *Mdr2^−^*^/*−*^ mice and decreases FXR/FGF15 signaling [55].

In general, GPBAR1 activation in immune cells negatively regulates inflammatory signaling. Both *Gpbar1^−^*^/*−*^ and FXR^−^^/*−*^ mice acquire a pro-inflammatory phenotype with age and exhibit an amplified inflammatory reaction when exposed to infectious agents. This implies that these receptors are components of the regulatory network that sustains tolerance of the hepatic immune system towards intestinal antigens and xenobiotics [4]. In the liver and intestine, GPBAR1 activation can promote macrophage polarization from a pro-inflammatory phenotype to an anti-inflammatory phenotype, suggesting BAs may serve as endogenous regulators of macrophage polarization [56,57]. Although monocyte-derived macrophages express both FXR and GPBAR1, it has been demonstrated that GPBAR1 is the dominant receptor in Kupffer cells (the resident macrophages of the liver) [58]. In the context of DCs and NKT cells, BAs induce DCs polarization toward a phenotype biased in favor of IL-12 and TNF-α via the GPBAR1-cAMP pathway [59]. Genetic depletion of GPBAR1 exacerbated liver damage in a mouse model of acute hepatitis and led to the recruitment of NKT1, a subtype of proinflammatory NKT cells [60]. On the other hand, overexpression of GPBAR1 rescues wild-type mice from acute hepatitis and induces the polarization of NKT cells to an anti-inflammatory phenotype (NKT10 subtype) [60]. In addition, genetic depletion of GPBAR1 significantly increased MC population along with decreased collagen deposition in a mouse model of autoimmune skin-related disease [61].

Interestingly, Urszula et al. have recently summarized the immunomodulatory effects and expression of major BA receptors in different immune cells, including monocytes, dendritic cells (DCs), B cells, T cells, granulocytes, and NK cells [49]. Most BA receptors can bind multiple ligands with different affinities. FXR exhibits binding affinities to BAs in descending order of: CDCA > DCA > LCA > CA > UDCA, isoDCA, whereas GPBAR1 is bound by BAs in the following order LCA > DCA > CDCA > CA > UDCA, TLCA [49,62]. Nevertheless, higher affinity does not always ensure more prominent effects. For example, isoDCA, no other BAs with higher affinities, limits FXR activation in DCs to promote differentiation of regulatory T cells (Tregs) cells [63]. More efforts are needed to compare the efficacy of various ligands and define the optimal affinities of these interactions in AILDs.

### 4.2. BAs and Immune Cells in Adaptive Immune System

**Figure 1:**
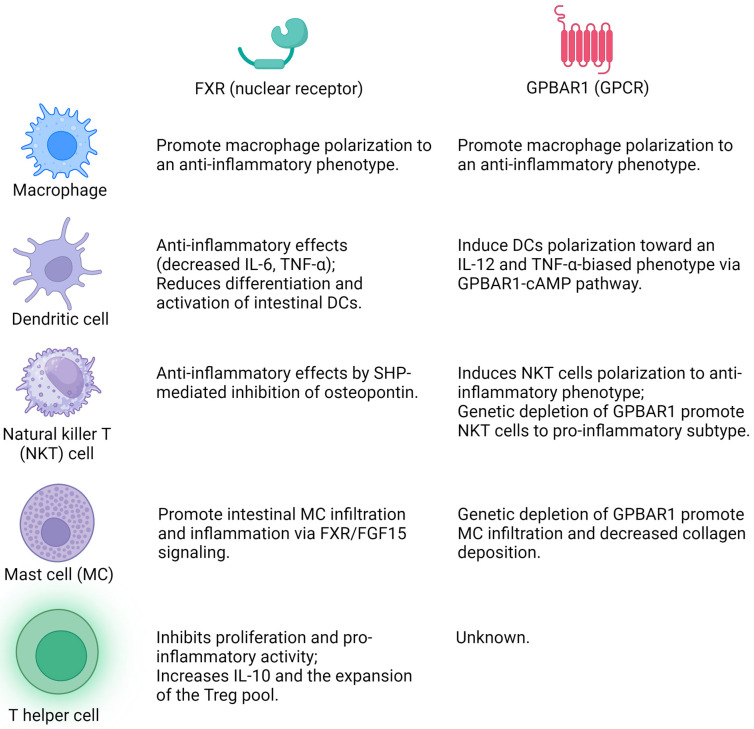
The effects of BA receptor (FXR and GPBAR1) agonist/antagonists on different immune cells (macrophage, dendritic cell, natural killer T cell, mast cell, and T helper cell). The figure was created with biorender.com.

In addition to the interactions with innate immune cells, BA signaling has been demonstrated to directly mediate adaptive immunity. Although T cells were traditionally considered to lack expression of both FXR and GPBAR1, recent research has also explored the essential roles of BAs via VDR and FXR in adaptive immunity of inflammatory bowel disease [63,64]. In T helper cells, stimulation of VDR and FXR inhibits their pro-inflammatory and proliferation actions, which increases IL-10 secretion and Treg population [50]. In addition, restoration of the intestinal BA pool (combination of 3-oxoLCA and LCA) can alleviate host susceptibility to inflammatory colitis in a VDR-dependent mechanism by enhancing a unique RORγ^+^ Treg population [64]. However, in relation to AILDs, the role of BAs in adaptive immunity remains unclear. Overall, it is worth acknowledging that the regulation role of BA metabolism on immune response may contribute to intestine–liver crosstalk and the development of cholangiopathies [4,65]. Understanding the impact of these diverse immune cells on BA signaling would aid in pinpointing potential therapeutic targets for AILDs (Figure 1).

## 5. Clinical Studies Focusing on BA Signaling Pathways for Treatment of AILDs

Basic research in BA signaling has been translated to therapies based on BAs for liver diseases including AILDs and nonalcoholic fatty liver disease (NAFLD), now termed metabolic dysfunction-associated steatotic liver disease (MASLD) [66,67]. An overview of recent studies conducted concerning AILDs can be reviewed in Table 1. Overall, the major strategies for AILDs related to BA signaling target BA receptors and BA transporters in the gut–liver axis as discussed below.

### 5.1. Pharmacological Activation of BA Receptors

For pharmacological activation of BA receptors, so far, agonists of FXR and GPBAR1 have attracted the most attention as drug targets.

OCA is a semi-synthetic BA and has been considered a steroidal FXR agonist. Phase III trials on OCA in PBC patients have shown its great potential as an effective therapeutic option with decreased serum marker levels and improvement of hepatic BA excretion [80,81]. Regarding PSC patients, a phase II trial revealed that 5–10 mg OCA reduced serum alkaline phosphatase (ALP) during an initial 24-week treatment period [68]. In a rat model of liver cirrhosis induced by CCl_4,_ the non-steroidal FXR agonist PX20606 ameliorates liver fibrosis and hepatic inflammation along with reduced serum alanine aminotransferase (ALT) and aspartate aminotransferase (AST) [82]. Cilofexor (GS-9674) is another non-steroidal FXR agonist that has undergone a phase II placebo-controlled study in PSC due to its anti-inflammatory and antifibrotic potential [69]. In another phase II study of Cilofexor, serum secretion of IL-31 and pruritus severity were increased in the MASLD population, whereas baseline levels of IL-31 were associated with pruritus in PSC and PBC patients [70]. A different non-steroidal FXR agonist, Tropifexor (LJN452), has been investigated under phase II trials in patients with PBC and MASLD, which caused a dose-dependent reduction in gamma–glutamyl transferase (GGT) and ALT levels [46,71,83]. EDP-305 is a steroidal and non-BA FXR agonist and has undergone a Phase II trial in patients with PBC, which showed significant improvement in serum levels of ALT, AST, and GGT [46]. BAR502 is a ligand for both FXR and GPBAR1 (slightly preferential for GPBAR1), which attenuates liver damage without inducing pruritus in mouse models of cholestasis [84]. Although many clinical trials on FXR agonists recruit patients with PBC and PSC, it is clear that MASLD draws the most attention due to its substantial market potential.

### 5.2. Pharmacological Modulation of BA Transporters

In the intestine, FXR activation induces expression of the intestinal hormone FGF15/19 (FGF15 in mice and FGF19 in humans). NGM282 (also known as FGF19-M70 or Aldafermin) is the only FGF19 analogue that has been tested under Phase II studies in patients with PBC, which shows reduced BA synthesis and serum levels of ALP, AST with no worsening of pruritus [72,73]. Regarding PSC patients, Phase II trials revealed that NGM282 suppressed BA synthesis and reduced fibrosis markers with no changes in serum ALP levels [74,85].

Cholangiocytes and ileal enterocytes both express ASBT, which plays an essential role in preserving BA homeostasis [43]. ASBT inhibitors have been shown to improve AILDs by enhancing the excretion of fecal BAs and subsequently increasing hepatic BA synthesis. In Phase II studies, Linerixibat (GSK2330672), an ASBT inhibitor, exhibited efficacy in decreasing the severity of pruritus in PBC. However, the long-term use of this drug could potentially be restricted due to the prevalent adverse event of diarrhea [75,76]. Odevixibat (A4250), another ASBT inhibitor, has undergone two phase II studies of PBC patients with pruritus, which remarkably improved pruritus along with decreased serum conjugated BAs and subsequently ~50% reduction in total BA concentrations [75,77]. However, in a separate Phase II study of PBC patients with pruritus, the ASBT inhibitor Maralixibat did not significantly ameliorate pruritus, maybe due to the abundant (47%) placebo effect [78]. Regarding PSC patients, a Phase II trial revealed that Maralixibat was associated with reduced serum BA levels along with improvement of pruritus [79]. Furthermore, a Phase II study on the ASBT inhibitor Volixibat is currently recruiting PSC patients with cholestatic pruritus.

## 6. Conclusions and Future Perspectives

In summary, BAs represent a dynamic category of mediators that exert bidirectional effects on different cell types within the gut–liver axis. Understanding the mechanisms that regulate this interaction poses a significant task for upcoming research, as basic questions remain unresolved. For example, how does the progression of AIH impair the flux of BAs from the liver to the gut? What is the most effective strategy in therapeutic settings (administration of BAs, modulation of BA transporters, or pharmacological activation of BA receptors)? Although undesirable side effects including severe pruritus continue to occur, clinical trials targeting BA receptors and BA transporters for the treatment of AILDs are quite promising. Combining drugs that focus on BA signaling with anti-itching agents targeting genes associated with pruritus may be a potential strategy to enhance the overall tolerability of treatment. Future studies on the development of BA receptor agonists specific to certain cell types are urgently needed for better therapeutic remedies for addressing AILDs.

## Figures and Tables

**Table 1:** Clinical Studies Focusing on BA Signaling Pathways for Treatment of AILDs.

Medication	Target Population	Dose	Trial Phase	Time	Endpoints	Results	Adverse Events	Ref.
OCA	N = 76 patients with PSC	1.5–3, 5–10 mg	Phase II	24 weeks	Primary: Serum ALP; Secondary: Hepatic function and biochemistry, fibrosis, FXR activity and disease-specific symptoms.	OCA 5–10 mg reduced serum ALP.	Pruritus.	[68]
Cilofexor (GS-9674)	N = 52 patients with PSC	30, 100 mg	Phase II	12 weeks	Primary: Safety; Exploratory efficacy endpoints: Serum ALP, GGT, ALT, AST, FGF19, C4, BAs, and liver fibrosis.	Improved liver biochemistries and markers of cholestasis.	Adverse events were similar between cilofexor and placebo-treated patients.	[69]
	N = 71 patients with PBC	30, 100 mg	Phase II	12 weeks	Primary: Safety; tolerability, markers of bile acid homestasis (serum C4, bile acids), liver biochemistry, and serum fibrosis markers	Improved serum liver biochemical tests.	The incidence of Grade 2 or 3 pruritus was higher with the 100 mg treatment group.	[70]
Tropifexor (LJN452)	N = 61 patients with PBC	Not reported	Phase II	12 weeks (Parts A and B)	Primary: GGT, blood pressure, pulse rate, body temperature, ECG, hemoglobin; Secondary: PK, PBC-40 score, pruritus.	Induced dose-dependent decline of GGT and ALT.	Not reported.	[71]
EDP-305	N = 68 patients with PBC	1, 2.5 mg	Phase II	12 weeks	Primary: Percentage of participants with at least a 20% reduction in ALP or normalization of ALP; Secondary: Adverse event, bilirubin, ALT, AST, GGT, liver fibrosis markers, CRP, haptoglobin and Alpha2 macroglobulin, TG, TC, HDL-C, LDL-C, domain and total scores, VAS, Cmax, Tmax, AUClast, FGF19, C4, BA.	EDP-305 decreased levels of ALT, AST and markers of cholestasis, but the primary endpoint of at least 20% reduction in ALT was not met.	Not reported.	[46]
NGM282 (FGF19-M70)	N = 36 patients with PBC	Not reported	Phase II	24 weeks	Primary: Plasma ALP; Secondary: Bilirubin, ALT, AST, and GGT.	Decreased the serum levels of C4 and ALP, inhibited BA synthesis.	Not reported.	[72]
	N = 45 PBC patients with incomplete response to ursodiol	0.3, 3 mg	Phase II	4 weeks	Primary: ALP; Secondary: Levels of ALT, AST, GGT, bilirubin, C4, BAs, ALP, and symptom of pruritus.	Reduced levels of ALP, transaminases and immunoglobulins.	Gastrointestinal disorders.	[73]
	N = 62 patients with PSC	1, 3 mg	Phase II	12 weeks	Primary: ALP; Secondary: Serum biomarkers of BA metabolism and fibrosis.	Inhibited BA synthesis and decreased fibrosis markers, without significantly affecting ALP levels.	Gastrointestinal symptoms.	[74]
Linerixibat (GSK2330672)	N = 22 patients with PBC	45, 90 mg	Phase II	2 weeks	Primary: Safety; Secondary: Pruritus scores, serum total BAs, C4, pharmacokinetic parameters of UDCA and its conjugates.	Improved pruritus, serum total and conjugated BAs.	Diarrhea.	[75]
	N = 147 patients with PBC	20–180 mg	Phase II	16 weeks	Primary: Mean Worst Daily Itch Score.	Improved pruritus.	Diarrhea, abdominal pain.	[76]
Odevixibat (A4250)	N = 9 patients with PBC	0.75, 1.5 mg	Phase II	4 weeks	Primary: Safety and tolerability; Secondary: Pruritus variables and quality of life.	Reduced serum BAs, improved pruritus and sleep disturbance.	Increased transaminases.	[75]
	N = 24 patients with PBC	10–200 μg/kg	Phase II	4 weeks	Primary: Serum BA levels; Secondary: VAS-itch, Whitington itch, and PO-SCORAD itch and sleep disturbance scores.	No significant improvements in pruritus.	Gastrointestinal disorders.	[77]
Maralixibat	N = 66 patients with PBC	10, 20 mg	Phase II	13 weeks	Primary: Adult ItchRO™; Secondary: Adult ItchRO™, fasting sBA levels, serum C4 levels, ALP levels and 5-D Itch scores.	No significant improvements in pruritus.	Gastrointestinal disorders.	[78]
	N = 27 patients with PSC	0.5–10 mg	Phase II	14 weeks	Primary: Serum BA levels; Secondary: Adult ItchRO score.	Reduced BAs levels, but not ALP and other liver biochemistries.	Diarrhea and other GI symptoms.	[79]
Volixibat	N = 200 patients with PSC	20, 80 mg	Phase II	28 weeks	Primary: Adult ItchRO; Secondary: Serum BA levels, ALT, AST, ALP, bilirubin, PROMIS^®^.	Recruiting.		

## Data Availability

Not applicable.

## Abbreviations

AE2: Cl^−^/HCO_3_^−^ anion exchanger 2; AIH, autoimmune hepatitis; AILD, autoimmune liver disease; ALP, alkaline phosphatase; ALT, alanine aminotransferase; AST, aspartate aminotransferase; ASBT, apical sodium-dependent bile acid transporter; cAMP, cyclic adenosine monophosphate; CA, Cholic acid; CDCA, Chenodeoxycholic acid; CYP7A1, cholesterol 7α-hydroxylase; CYP7B1, 25-Hydroxycholesterol 7α-hydroxylase; CYP27A1, Sterol 27-hydroxylase; DC, dendritic cell; DCA, deoxycholic acid; FXR, farnesoid X receptor; FGF, fibroblast growth factor; GPBAR1, G protein bile acid activated receptor 1; IBABP, ileal bile acid-binding protein; IgG, immunoglobulin G; IL-, interleukin-; LCA, lithocholic acid; MC, mast cell; MCA, muricholic acid; MRCP, magnetic resonance cholangiopancreatography; MRP3, multidrug resistance-associated protein 3; NTCP, Na^+^-taurocholate co-transporting polypeptide; OATP, organic anion transporting polypeptide; OCA, obeticholic acid; OST, organic solute transporter; pANCA, perinuclear staining pattern; PBA, primary bile acid; PBC, primary biliary cholangitis; PDC-E2, E2 component of mitochondrial pyruvate dehydrogenase complex; PSC, primary sclerosing cholangitis; SHP, small heterodimer partner; TGR5, Takeda G protein coupled receptor 5; TMEM16A, transmembrane member 16A; TNF-α, tumor necrosis factor alpha; TNFAIP3, tumor necrosis factor-α inducible protein 3; Tregs, regulatory T cells; UDCA, ursodeoxycholic acid; UTI, urinary tract infection.
